# An exploratory, large-scale study of pain and quality of life outcomes in cancer patients with moderate or severe pain, and variables predicting improvement

**DOI:** 10.1371/journal.pone.0193233

**Published:** 2018-04-03

**Authors:** Constanza Maximiano, Iker López, Cristina Martín, Luis Zugazabeitia, Juan L. Martí-Ciriquián, Miguel A. Núñez, Jorge Contreras, Michael Herdman, Susana Traseira, Mariano Provencio

**Affiliations:** 1 Hospital Puerta de Hierro de Majadahonda, Medical Oncology, Madrid, Spain; 2 Hospital Universitario Virgen del Rocío, Medical Oncology, Sevilla, Spain; 3 Hospital de la Santa Creu i Sant Pau, Medical Oncology, Barcelona, Spain; 4 Hospital Povisa, Radiation Oncology, Pontevedra, Spain; 5 Hospital General Universitario de Alicante, Medical Oncology, Alicante, Spain; 6 Hospital Santa María Nai, Medical Oncology, Ourense, Spain; 7 Complejo Hospitalario Regional Carlos Haya, Radiation Oncology, Málaga, Spain; 8 Insight Consulting and Research, Barcelona, Spain; 9 Mundipharma Pharmaceuticals S.L., Medical Department, Madrid, Spain; Stanford University School of Medicine, UNITED STATES

## Abstract

**Background:**

There have been few large-scale, real world studies in Spain to assess change in pain and quality of life (QOL) outcomes in cancer patients with moderate to severe pain. This study aimed to assess changes on both outcomes after 3 months of usual care and to investigate factors associated with change in QoL.

**Patients and methods:**

Large, multi-centre, observational study in patients with lung, head and neck, colorectal or breast cancer experiencing a first episode of moderate to severe pain while attending one of the participating centres. QoL was assessed using the EuroQol-5D questionnaire and pain using the Brief Pain Inventory (BPI). Instruments were administered at baseline and after 3 months of follow up. Multivariate analyses were used to assess the impact of treatment factors, demographic and clinical variables, pain and other symptoms on QoL scores.

**Results:**

1711 patients were included for analysis. After 3 months of usual care, a significant improvement was observed in pain and QoL in all four cancer groups (p<0.001). Effect sizes were medium to large on the BPI and EQ-5D Index and Visual Analogue Scale (VAS). Improvements were seen on the majority of EQ-5D dimensions in all patient groups, though breast cancer patients showed the largest gains. Poorer baseline performance status (ECOG) and the presence of anxiety/depression were associated with significantly poorer QOL outcomes. Improvements in BPI pain scores were associated with improved QoL.

**Conclusion:**

In the four cancer types studied, pain and QoL outcomes improved considerably after 3 months of usual care. Improvements in pain made a substantial contribution to QoL gains whilst the presence of anxiety and depression and poor baseline performance status significantly constrained improvement.

## Introduction

Pain is a common and burdensome symptom in cancer patients [1] with data indicating that 50%–90% will require treatment for pain during the course of their disease [2]. Opioids are recommended for the management of moderate/severe cancer pain by the World Health Organization and current guidelines [3, 4] and are recognized as the treatment of choice in these patients [5]. Despite the importance of adequate pain management, however, studies show that there is substantial undertreatment of cancer pain [6 Deandrea]. One of the causes for this undertreatment could be the underuse of pain scales by health professionals in clinical practice.

Pain is clearly an important, specific outcome to monitor when assessing the results of cancer patient management in routine care. Quality of life on the other hand is a broad, multidimensional concept and a very relevant outcome for patients. In terms of monitoring the broader outcomes of care, studies have shown that health-related quality of life (HRQoL) questionnaires are a practical tool for this purpose [7]. To date, there have been few large-scale studies in Spain to describe real world outcomes over time in cancer patients with moderate to severe pain. Such studies are important because they show how pain outcomes evolve in conditions of usual care, in patients who are usually much more heterogenous than those included in clinical trials and where the presence of co-morbidites, lifestyle factors, and poly-medication can all potentially impact results.

Standardised assessment of pain and QOL in routine care can also help to identify patients who are at risk of poorer outcomes. Previous studies have identified various determinants of QoL in cancer patients including age, pain, appetite loss, fatigue, intestinal function, performance status, emotional functioning, presence of metastases, and treatment status [8–10]. However, earlier studies relied largely on cross-sectional data and relatively few have looked at the association between specific patient and treatment-related variables and change in QOL.

The C2 study was a large, exploratory study which aimed to assess changes in pain and QOL in cancer patients with moderate-severe cancer pain after 3 months of usual care and to investigate factors associated with change in QoL. Changes in pain and QOL were assessed and estimated using effect sizes and multivariate regression models were constructed to investigate prognostic variables predicting change in QOL. In this paper, we report results from four of the most commonly diagnosed cancers (lung, breast, colorectal, and head and neck). Although the study was performed in Spain, the large number and heterogeneity of patients included and the investigation of factors contributing to change in pain and QoL outcomes should mean that the study is relevant to researchers more generally who are interested in pain and quality of life outcomes in clinical practice in cancer patients.

## Materials and methods

### Design

The C2 study was a multi-center, prospective, longitudinal, observational study carried out in 150 oncology units throughout Spain. The present analysis was performed in a subset of the patients included in the study, i.e. patients with lung, breast, colorectal, or head and neck cancer. The C2 study protocol was approved by the Ethics Committee of the coordinating hospital “Hospital Universitario Puerta de Hierro Majadahonda”, file number 266. All patients provided written informed consent to participate.

### Sample

Patients were eligible for enrolment in the C2 study if they were ≥18 years or older and experiencing moderate to severe pain (measured using a Numeric Rating Scale, NRS_0-10_ ≥4) for the first time while attending one of the participating oncology units. Patients were screened consecutively for enrolment during routine clinic visits. The patients included were predominantly managed in an out-patient setting. Patients were excluded if they had psychiatric or neurological disorders which, in the investigator’s judgement, meant they were unable to complete the study materials. Patients with stable psychiatric conditions who were able to complete the study questionnaires were eligible for inclusion.

A total of 2643 patients covering 21 different types of cancer were included in the C2 Study between June 2011 and July 2012. As the aim of the study was to describe pain and QOL outcomes in a broad cross-section of the patient population with moderate to severe pain treated in oncology units, a wide range of such centres was included from large teaching hospitals to small country hospitals, and including urban and rural settings in all regions of Spain. For the purposes of the present analysis, we focussed on the 1711 patients with lung, head and neck, colorectal, or breast cancer. These were the 4 most prevalent cancers in the study population and each represented over 10% of the total sample. Of the 17 cancer types excluded from the present analysis, the most common types were prostate (n = 222) and pancreas (n = 109) and the least frequent were brain (n = 7) and thyroid (n = 6).

As the C2 Study was designed as an exploratory study, information on study refusal and screening rejection rates was not collected though in these types of study in Spain the refusal rate is normally low. Of the patients included at baseline, 19.5% (488 patients) did not attend the 3 month visit, most commonly because of loss to follow-up (n = 377, or 77.3% of those who did not complete the study) and withdrawal of informed consent (n = 74, 15.2%). Thirty-four patients (7%) were discharged and 2 patients died.

### Sample size calculations

Sample size for the C2 study as a whole was calculated based on determining the number of subjects required to assess the prognostic factors affecting evolution in QOL assessed using the EQ-5D questionnaire. For the purposes of this study, we defined an improvement in QoL as an increase ≥30% in EQ-5D Index and we initially considered 17 potential prognostic factors that meant individuals could be classified into one of 17 different distributions of values for these variables. After incorporating assumptions regarding the number of patients who would improve and the proportion of variance in the model that would be explained by the prognostic factors included and using an alpha error of 0.05 and a statistical power of 0.8, the final sample size for the C2 study as a whole was n = 3,008.

### Procedures

Patients were followed for 3 months from study inclusion and made a total of 3 study visits (baseline, 1 month, and 3 months). Pain and QoL were assessed at baseline and 3 months. The 1 month visit was used to collect data on aspects such as dose changes, intestinal function and pain, but data on QOL was not collected, so results from this visit are not included in the present analysis. Surveys were completed in person during routine clinic visits with support form health care personnel if needed. Participating centres followed their usual approach to patient management in all cases.

### Measures

Pain was categorised using the original version of the Edmonton Classification System for Cancer Pain (ECS-CP) [11]. Pain severity and impact were assessed using the short form of the Brief Pain Inventory (BPI-SF) [12], which was recently validated for use in Spain [13]. The BPI-SF measures patient perceptions of pain severity and degree of pain interference on daily functioning and provides sub-scale scores for each dimension ranging from 0 (no pain or interference) to 10 (maximum pain or interference).

Quality of life was measured using the EQ-5D questionnaire [14] which measures QoL in 5 dimensions (mobility, self-care, usual activities, pain/discomfort, anxiety/depression). Each dimension has three response levels (no problems, some problems, extreme problems) and respondents check one level on each dimension to indicate their health ‘today’. A single summary score (EQ-Index) is provided for each health state defined by the instrument based on societal values (utility weights). In the present study, we used Spanish utility weights, which range from -0.0757 for the poorest health state to 1 for the best state [15]. Respondents also rated their overall health status on a 0–100 visual analogue scale (EQ-VAS) where 0 represents the worst and 100 the best imaginable health state. Both the EQ-5D and BPI-SF were self-administered.

Other data collected included age, sex, primary tumour site, functional status (measured using the Eastern Cooperative Oncology Group performance status [ECOG]), time since diagnosis, presence and site of metastases, pain origin (tumour, metastases, antineoplastic treatments, other), intestinal function (measured with the Bowel Function Index–BFI- [16]), and treatment.

### Analysis

Change over time was assessed using paired t-tests or Wilcoxon tests. Effect sizes (ES) were calculated to help interpret the magnitude of change. An ES of 0.20 was considered to represent a small change, 0.50 a moderate change, and 0.80 a large change [17]. Multivariate regression models were constructed to analyze the degree to which different prognostic variables predicted the magnitude of change in overall QOL assessed using the EQ-5D Index. The analysis was performed for each of the four types of cancer studied. We initially assessed 21 potential prognostic variables which were initially excluded based on bivariate analyses, using a cut-off of p>0.2. Variables that remained after bivariate analysis were then included independently in the regression model using forward and backward stepwise analysis until the final model was derived. The dependent variable was change on the EQ-5D Index. A further analysis was carried out using multivariate logistic regression to test which factors might be associated with a patient reporting extreme pain at month 3. The dependent variable was ‘extreme pain’ at month 3 with age, sex, and ECOG status, presence of neuropathic pain, and emotional distress at baseline selected as potential independent variables for inclusion in the model. Patients with any of the 4 types of cancer were included in the analysis. Preliminary bivariate analysis was performed to test for association between the independent and dependent variables and only those independent variables with a p-value of <0.2 for association were included. SPSS (version 17) was used for all analyses and results were considered statistically significant at p<0.05.

## Results

The demographic, clinical and treatment characteristics of the study population included for the current analysis are shown in Table 1. In all cancer types, the majority of patients were in ECOG 0 or 1 (range from 63.2% in colorectal cancer to 75.2% in head and neck cancer). Almost 70% of patients were on chemotherapy, which was largely palliative, except in head and neck cancer (72.7% curative).

**Table 1:** Descriptive analysis showing baseline socio-demographic, clinical and treatment characteristics of the study population by cancer type.

Variable	Lung n (%) 706 (41.3)	Head and neck n (%) 363 (21.2)	Colorectal n (%) 334 (19.5)	Breast n (%) 308 (18.0)
**Socio-demographic**				
Age, years, mean (SD) [range]	63.3 (10.1) [33–86]	61.7 (11.7) [24–88]	66 (11.7) [25–91]	61 (11.7) [26–89]
Sex, n (%)				
Male	558 (79)	286 (79)	204 (61)	10 (3)
Female	148 (21)	77 (21)	130 (39)	298 (97)
**Clinical**				
Functional status				
*ECOG 0*	21 (3)	88 (24.2)	20 (6)	18 (5.8)
*ECOG 1*	434 (61.5)	185 (51)	191 (57.2)	188 (61.0)
*ECOG 2*	218 (30.9)	78 (21.5)	86 (25.7)	87 (28.2)
*ECOG 3*	28 (4)	9 (2.5)	33 (9.9)	15 (4.9)
*ECOG 4*	4 (0.6)	3 (0.8)	2 (0.6)	0 (0)
Time since diagnosis, mean (SD) and [range], in months	8.1 (10.7) [0–84]	10.5 (19.1) [0–216]	20.7 (25.7) [0–204]	50.7 (58) [0–480]
Metastases	565 (80)	124 (34.2)	257 (76.9)	243 (78.9)
Pain secondary to				
*Primary tumour*	283 (40.1)	191(52.6)	126 (37.7)	32 (10.4)
*Metastases*	384 (54.4)	44 (12.1)	177 (53.0)	221 (71.8)
*Antineoplastic treatment*	61 (8.6)	184 (50.7)	41 (12.3)	36 (11.7)
*Not related to cancer*	70 (9.9)	23 (6.3)	50 (15.0)	42 (13.6)
Edmonton classification system for cancer pain (ECS-CP)				
*Visceral*, *bone or soft tissue pain*	421 (59.6)	209 (57.6)	228 (68.3)	241 (78.2)
*Neuropathic pain*, *mixed or unknown*	210 (29.7)	122 (33.6)	117 (35.0)	142 (46.1)
*Incident pain*	170 (24.1)	124 (34.2)	84 (25.1)	43 (14.0)
*Psychological distress*	115 (16.3)	64 (17.6)	67 (20.1)	48 (15.6)
*Slow opioid escalation*	84 (11.9)	63 (17.4)	56 (16.8)	60 (19.5)
Intestinal function				
*BFI*^*1*^ *≤ 30*	518 (73.4)	275 (75.8)	200 (59.9)	227 (73.7)
*BFI > 30*	159 (22.5)	81 (22.3)	123 (36.8)	64 (20.8)
**Treatment**				
Chemotherapy	512 (72.5)	245 (67.5)	234 (70.1)	201 (65.3)
*Palliative*	412 (80.5)	66 (26.9)	173 (73.9)	170 (84.6)
*Curative*	100 (19.5)	178 (72.7)	61 (26.1)	31 (15.4)
Treatment line				
*1*	341 (66.6)	202 (82.4)	126 (53.8)	75 (37.3)
*2*	126 (24.6)	33 (13.5)	60 (25.6)	68 (33.8)
*3*	34 (6.6)	7 (2.9)	26 (11.1)	27 (13.4)
*> 3*	11 (2.1)	2 (0.8)	22 (9.4)	31 (15.4)
Patients receiving radiotherapy at baseline	282 (39.9)	268 (73.8)	96 (28.7)	127 (41.2)
*Palliative*	198(70.2)	38 (14.2)	52 (54.2)	107 (84.3)
*Curative*	84(29.8)	230 (85.8)	44 (45.8)	20 (15.7)
Pain treatment (WHO scale)	694 (98.3)	357 (98.3)	334 (100)	306 (99.4)
*Step 1*: *non-opioid+optional adjuvant*	363 (52.3)	219 (61.3)	212 (63.5)	157 (51.3)
*Step 2*: *weak opioid + non-opioid + optional adjuvant*	96 (13.8)	41 (11.5)	35 (10.5)	41 (13.4)
*Step 3*: *strong opioid + non-opioid + optional adjuvant*	548 (79)	287 (80.4)	251 (75.1)	224 (73.2)
Antiemesis/laxatives				
*Laxative*	223 (32.1)	106 (29.7)	124 (37.1)	97 (31.7)
*Antiemetic*	152 (21.9)	64 (17.9)	108 (32.3)	69 (22.5)

^1^BFI, Bowel Function Inventory.

Change in BPI scores from baseline to 3-month follow-up are shown in Table 2. Across all groups, the severity summary score improved from an overall mean (SD) of 5.3 (1.6) to 2.3 (1.9) and the interference summary score from 5.2 (2.1) to 2.8 (2.3). All cancer sub-types showed considerable improvement, with large effect sizes ranging from 1.59 for the colorectal group to 2.13 for head and neck cancer patients on the BPI severity sub-score and from 1.0 for the lung cancer patients to 1.43 for the breast cancer group on the interference sub-scale.

**Table 2:** Descriptive analysis showing scores on the Brief Pain Inventory by cancer type at baseline and 3 months, means and standard deviations.

	Lung		Head and neck		Colorectal		Breast	
	Baseline	3-month follow-up	Baseline	3-month follow-up	Baseline	3-month follow-up	Baseline	3-month follow-up
**Pain severity (0–10)**								
Maximum pain intensity	7 (1.6)	3.5 (2.5)	7.2 (1.5)	3.3 (2.3)	6.9 (1.8)	3.7 (2.8)	7.0 (1.6)	3.5 (2.2)
Minimum pain intensity	3.7 (2.2)	1.5 (1.6)	3.3 (2.0)	1.1 (1.4)	3.5 (2.1)	1.5 (1.9)	4.4 (2.5)	1.8 (1.8)
Average pain intensity	5.4 (1.6)	2.4 (1.9)	5.3 (1.6)	2.2 (1.8)	5.3 (1.8)	2.4 (2.2)	5.7 (1.8)	2.6 (1.9)
Pain intensity at the time of completion	5.2 (2.1)	2.0 (2.0)	5.0 (2.1)	1.6 (1.8)	4.8 (2.3)	2.0 (2.2)	5.5 (2.2)	2.3 (1.9)
Severity summary score	5.3 (1.6)	2.3 (1.9)	5.2 (1.5)	2.0 (1.7)	5.1 (1.7)	2.4 (2.1)	5.6 (1.8)	2.5 (1.8)
**Pain interference (0–10)**								
General activity	5.9 (2.3)	3.4 (2.8)	5.4 (2.3)	2.8 (2.4)	6.2 (2.3)	3.5 (3.0)	6.2 (2.1)	3.1 (2.3)
Mood	5.4 (2.5)	3.1 (2.9)	5.7 (2.5)	2.7 (2.4)	5.6 (3.0)	3.4 (3.1)	6.1 (2.5)	2.9 (2.5)
Walking	3.9 (3.0)	2.5 (2.8)	2.0 (2.6)	1.0 (1.7)	4.7 (3.0)	2.7 (2.7)	4.9 (3.0)	2.3 (2.2)
Usual work	5.6 (2.5)	3.4 (2.9)	4.6 (2.9)	2.6 (2.5)	6.1 (2.5)	3.7 (3.2)	6.2 (2.4)	3.1 (2.5)
Relations with others	4.3 (2.7)	2.5 (2.6)	5.0 (2.6)	2.6 (2.5)	4.5 (3.1)	2.6 (2.7)	5.0 (2.8)	2.5 (2.3)
Sleep	4.4 (2.9)	2.1 (2.3)	4.7 (2.9)	2.2 (2.4)	4.7 (3.1)	2.5 (2.6)	5.1 (2.9)	2.2 (2.2)
Enjoyment	5.8 (2.5)	3.5 (3.0)	6.1 (2.3)	3.1 (2.6)	6.4 (2.6)	3.9 (3.2)	6.2 (2.4)	3.0 (2.5)
Interference summary score	5.0 (2.1)	2.9 (2.5)	4.8 (1.9)	2.4 (2.1)	5.5 (2.2)	3.2 (2.7)	5.7 (2.1)	2.7 (2.1)

All baseline to 3 month changes were statistically significant at <0.001 (Wilcoxon test).

Changes in QoL are shown in Table 3. All cancer types showed improvements on all dimensions of EQ-5D, though they were particularly marked in breast cancer patients. In all cases, the greatest improvements were seen on the pain/discomfort dimension of EQ-5D with, for example, 39.2% of lung cancer patients reporting no pain/discomfort after 3 months of follow-up compared to only 1.6% at baseline. The reduction in the proportion of patients reporting extreme pain/discomfort was also notable (e.g. from 42.5% of breast cancer patients at baseline to only 6.1% at 3 months). Improvements on EQ-5D dimensions were reflected in changes on the EQ-Index, with improvements ranging from 0.12 points (on a scale from -0.0757 to 1) in lung cancer patients to 0.25 in breast cancer patients. Similar magnitudes of change were seen on the EQ-VAS, though interestingly breast cancer patients showed the smallest change there with a gain of 10.3 points on a scale of 0–100. The changes observed correspond to moderate to large ES.

**Table 3:** Results of bivariate analysis showing changes in quality of life (EuroQoL-5D) by cancer type from baseline to 3-month follow-up visit.

	Lung N = 495			Head and neck N = 315			Colorectal N = 271			Breast N = 280		
	Baseline	3-month follow-up	*P*	Baseline	3-month follow-up	*p*	Baseline	3-month follow-up	*p*	Baseline	3-month follow-up	*p*
**Dimensions, n (%)**												
*Mobility*												
No problems	224 (45.3)	274 (55.4)	*<0*.*001*^*1*^	252 (80.0)	262 (83.2)	*0*.*469*^*1*^	116 (42.8)	142 (52.4)	*<0*.*001*^*1*^	103 (36.8)	152 (54.3)	*<0*.*001*^*1*^
Some problems	248 (50.1)	177 (35.8)		59 (18.7)	48 (15.2)		135 (49.8)	105 (38.7)		153 (54.6)	120 (42.9)	
Confined to bed	23 (4.6)	44 (8.9)		4 (1.3)	5 (1.6)		20 (7.4)	24 (8.9)		24 (8.6)	8 (2.9)	
*Self-care*												
No problems	242 (48.9)	294 (59.4)	*<0*.*001*^*1*^	211 (67.0)	222 (70.5)	*0*.*367*^*1*^	154 (56.8)	161 (59.4)	*0*.*371*^*1*^	102 (36.4)	182 (65.0)	*<0*.*001*^*1*^
Some problems	234 (47.3)	156 (31.5)		95 (30.2)	87 (27.6)		95 (35.1)	84 (31.0)		145 (51.8)	88 (31.4)	
Unable to	19 (3.8)	45 (9.1)		9 (2.9)	6 (1.9)		22 (8.1)	26 (9.6)		33 (11.8)	10 (3.6)	
*Usual activities*												
No problems	110 (22.2)	184 (37.2)	*<0*.*001*^*1*^	91 (28.9)	142 (45.1)	*<0*.*001*^*1*^	50 (18.5)	102 (37.6)	*<0*.*001*^*1*^	33 (11.8)	110 (39.3)	*<0*.*001*^*1*^
Some problems	335 (67.7)	235 (47.5)		194 (61.6)	156 (49.5)		164 (60.5)	114 (42.1)		185 (66.1)	148 (52.9)	
Unable to	50 (10.1)	76 (15.4)		30 (9.5)	17 (5.4)		57 (21.0)	55 (20.3)		62 (22.1)	22 (7.9)	
*Pain / discomfort*												
None	8 (1.6)	194 (39.2)	*<0*.*001*^*1*^	9 (2.9)	125 (39.7)	*<0*.*001*^*1*^	1 (0.4)	115 (42.4)	*<0*.*001*^*1*^	3 (1.1)	122 (43.6)	*<0*.*001*^*1*^
Moderate	337 (68.1)	260 (52.5)		160 (50.8)	169 (53.7)		154 (56.8)	126 (46.5)		158 (56.4)	141 (50.4)	
Extreme	150 (30.3)	41 (8.3)		146 (46.3)	21 (6.7)		116 (42.8)	30 (11.1)		119 (42.5)	17 (6.1)	
*Anxiety/ depression*												
None	193 (39.0)	250 (50.5)	*<0*.*001*^*1*^	94 (29.8)	175 (55.6)	*<0*.*001*^*1*^	88 (32.5)	128 (47.2)	*<0*.*001*^*1*^	74 (26.4)	160 (57.1)	*<0*.*001*^*1*^
Moderate	269 (54.3)	217 (43.8)		175 (55.6)	120 (38.1)		138 (50.9)	108 (39.9)		166 (59.3)	103 (36.8)	
Extreme	33 (6.7)	28 (5.7)		46 (14.6)	20 (6.3)		45 (16.6)	35 (12.9)		40 (14.3)	17 (6.1)	
**Index score**^**3**^ mean (SD)	0.51 (0.22)	0.63 (0.29)	*<0*.*001*^*2*^ (0.55)*	0.51 (0.21)	0.72 (0.23)	*<0*.*001*^*2*^ *(1*.*0)*	0.45 (0.25)	0.61 (0.31)	*<0*.*001*^*2*^ *(0*.*64)*	0.41 (0.24)	0.66 (0.22)	*<0*.*001*^*2*^ *(1*.*04)*
**EQ-5D VAS** (0–100)^4^ mean (SD)	47.7 (20.0)	59.2 (23.8)	*<0*.*001*^*2*^ *(0*.*58)*	49.3 (18.8)	65.7 (18.4)	*<0*.*001*^*2*^ *(0*.*87)*	47.1 (20.8)	58.3 (25.9)	*<0*.*001*^*2*^ *(0*.*54)*	44.0 (24.2)	54.3 (28.8)	*<0*.*001*^*2*^ *(0*.*43)*

^1^ Chi-square test.

^2^ Fisher’s exact test.

^3^ EQ-5D Index score: ranges from -0.0757 (worst health state on EQ-5D descriptive system) to 1 (perfect health).

^4^ EQ-5D VAS scores ranges from 0 to 100, with higher scores indicating better QoL.

*Effect sizes are provided in parenthesis for continuous variables.

The bivariate and stepwise selection process for prognostic variables eliminated 9 of the 21 variables originally tested leading to a total of 12 variables included in the final regression models, though not all of those were statistically significant for the four cancer types investigated.

The variables most commonly associated with change in QoL (Table 4) were baseline ECOG score, change on BPI severity and interference sub-scales, baseline EQ-5D Index score, and presence of anxiety/depression at both visits. A reduction in BPI scores (i.e. less pain), particularly on the severity sub-scale, was associated with gains in QOL. On the other hand, poorer baseline performance status and the presence of self-reported anxiety/depression at both visits significantly constrained improvement in QoL. The coefficient representing the EQ-Index at baseline indicates that those with poorer baseline health status generally experienced larger improvements over the study period. Models showed good explanatory power with adjusted R^2^ of 0.597, 0.699, 0.644, and 0.815, respectively, for lung, head and neck, colorectal, and breast cancer.

**Table 4:** Factors predicting change in QoL measured by the EQ-5D Index: Multivariate regression analyses.

	LUNG					HEAD AND NECK					COLORECTAL					BREAST				
				95% CI					95% CI					95% CI					95% CI	
	*B*	B	*p* value	LCI	UCI	*β*	B	*p* value	LCI	UCI	*β*	B	*P* value	LCI	UCI	*β*	B	*p* value	LCI	UCI
**(Intercept)**		.319	.000	.172	.467		.543	.000	.426	.661		.285	.001	.121	.450		.378	.000	.192	.563
**Sex** (ref. female)						.072	.043	.025	.006	.081										
**Time since diagnosis (months)**											.133	.002	.012	.000	.003					
**ECOG 1** (ref. ECOG0)	-.184	-.109	.057	-.221	.003	-.142	-.071	.001	-.111	-.031						-.074				
**ECOG 2** (ref. ECOG0)	-.213	-.134	.026	-.252	-.016	-.152	-.095	.000	-.147	-.043						-.15				
**ECOG 3 or 4** (ref. ECOG0)	-.152	-.188	.012	-.334	-.041	-.077	-.115	.021	-.213	-.018						-.137	-.244	.004	-.411	-.077
**Lung metastasis**											-.104	-.063	.024	-.117	-.008	-.071	-.051	.024	-.096	-.007
**Palliative chemotherapy**											.184	.117	.014	.024	.209					
**Curative chemotherapy**											.123	.140	.028	.015	.266					
**Line of treatment**^**1**^											-.246	-.062	.001	-.097	-.026					
**Anxiety/depression (EQ-5D dimension**^**2**^**)**	-.193	-.116	.000	-.169	-.064	-.279	-.143	.000	-.179	-.108	-.193	-.112	.000	-.173	-.052	-.196	-.117	.000	-.156	-.079
**EQ-5D Index at baseline**	.062	-.528	.000	-.650	-.407	-.567	-.683	.000	-.775	-.590	-.376	-.444	.000	-.591	-.296	-.577	-.697	.000	-.800	-.594
**Average pain at baseline**						-.192	-.031	.000	-.046	-.016	-.142	-.024	.023	-.044	-.003	-.149	-.024	.001	-.038	-.010
**Changes in BPI severity summary score**	-.479	-.055	.000	-.067	-.044	-.378	-.046	.000	-.059	-.033	-.353	-.045	.000	-.065	-.025	-.257	-.037	.000	-.052	-.022
**Changes in BPI interference summary score**		-.020	.003	-.033	-.007	-.258	-.035	.000	-.048	-.023	-.367	-.052	.000	-.072	-.031	-.318	-.049	.000	-.066	-.033
**ECS-CP**^**3**^																.112	.174	.000	.080	.269
**BFI Index**^**4**^	.087	.001	.030	.000	.002															

^**1**^Number of lines of treatment (1 = first, 2 = second, 3 = third, 4 = more than third) (reference = 0).

^**2**^ Anxiety/depression problems: 1 = present both at baseline and 3 months, 0 = without anxiety/depression problems at least in one visit (ref. = 0).

^**3**^ Edmonton: 1 = Poor prognosis; 0 = Good prognosis (ref. = 0).

^4^BFI Index: Intestinal functioning index (average of ease, sensation and personal judgment) (ref, BPI≤30, non-altered).

LCI = lower confidence interval; UCI = upper confidence interval.

Empty cells indicate non-statistically significant coefficients in each model.

In the logistic regression model to test for factors associated with reporting extreme pain at month 3, only being in ECOG performance status 3–4 at baseline was statistically significant. Age and sex were not included in the model as they failed to meet the criterion for inclusion at the stage of bivariate testing and neuropathic pain and emotional distress at baseline, though included, were not statistically significant.

## Discussion

In this exploratory study of outcomes in cancer patients treated in usual clinical practice in Spain, we observed substantial improvements in pain and QOL in the four cancer types studied. In terms of results on the pain measure (BPI), patients in all 4 groups showed similar magnitudes of improvement on the severity and interference sub-scales. The smallest improvement on the severity sub-scale was seen in the colorectal group, though the difference was small (change of 2.7 vs a mean change of 3.1 in the other three groups). All of the changes correspond to large effect sizes, indicating substantial improvement in pain in all four groups. As regards QOL, breast cancer patients showed the biggest improvement on the EQ-5D Index, followed by head and neck, colorectal, and lung cancer patients. Interestingly, the order was not the same on the EQ-5D VAS, where head and neck patients showed the biggest gains, followed by lung, colorectal, and breast cancer patients.

Discrepancies between the EQ-Index and EQ-VAS have been reported previously [18] and may be due to the fact that whereas the EQ-Index summarises results on the 5 specific health dimensions in the EuroQol descriptive system, the EQ-VAS asks patients to evaluate their overall health without reference to any specific dimensions of health. Similarly, the EQ-Index is calculated using general population utility weights, which could potentially affect results due to differential weighting by dimension, whilst the VAS is a simple self-rating of the patient’s health.

In regard to the head and neck cancer patients, the majority had pain secondary to the tumor or to treatment and were receiving curative treatment for localized cancer. Of note, these patients frequently show acute treatment-related toxicity including moderate to severe pain from surgery, mucositis related with radiotherapy, and drug-related neurotoxicity [19, 20]. Some of the improvement in pain and QOL observed in these patients may have been due to withdrawal or completion of radiotherapy over the study period.

While improvements were observed in all four patient groups and in practically all EQ-5D dimensions, after 3 months of follow-up varying proportions of patients still reported extreme problems in EQ-5D dimensions. Notably, 15.4% of lung cancer patients and 20.3% of colorectal cancer patients reported being unable to perform their usual activities, 11.1% of colorectal patients still reported extreme pain/discomfort, and 12.9% of the same group reported extreme anxiety/depression. Patients reporting this level of problem on any EQ-5D dimension would presumably form a priority group for further investigation and potentially for more concerted clinical action. In the analysis to test for factors associated with reporting extreme pain at month 3, only being in ECOG performance status 3–4 at baseline was statistically significant. This result is coherent as the overall deterioration in health status manifested by the ECOG rating is associated with more advanced disease or greater morbidity and/or toxicity and part of that clinical picture would be the presence of higher levels of pain. These results also suggest that ECOG performance ratings can be used as a prognostic factor. An earlier study by Bradley et al [21] also found that patients with poorer performance status had significantly higher symptom distress scores for a range of cancer-related symptoms, though in that case they used the Karnofsky rating scale.

In terms of the factors associated with change in QoL in all types of cancer, the most relevant were baseline performance status, change on the BPI severity and interference sub-scales, EQ-5D Index score at baseline, and presence of anxiety/depression at both visits. An earlier study demonstrated the relationship between improvements in pain as measured by the BPI and improvements in QoL [22], though that was not performed in cancer patients. The effect of changes in pain on QoL scores is strong, with the regression analysis suggesting that an approximately two point change on either the BPI pain severity or pain interference sub-scales would lead to a change on the EQ-Index which would be close to its minimal important difference (MID), i.e. the smallest difference in score on a scale which patients perceive as beneficial [23]. Research in cancer patients has shown that the MID for the EQ-Index ranges from 0.05 to 0.12 when using US and UK utility weights [24].

The regression analysis also showed that the presence of anxiety/depression substantially limited any gains in QoL. For example, based on the results of that analysis, colorectal cancer patients reporting anxiety/depression at baseline and at the final visit would have a much smaller improvement on the EQ-5D Index than those without anxiety/depression. The size of the difference (0.143 points) indicates that the presence of anxiety/depression likely affects several EQ-5D dimensions. These results are in line with others reported previously [25] and suggest that appropriate management of anxiety and depression in these patients could have substantial benefits in terms of overall QoL. With respect to the model, there was a degree of construct overlap between some of the predictor (pain, anxiety/depression) and outcome variables, i.e. between pain and anxiety/depression in the first case and QOL assessed using EQ-5D, which has pain/discomfort and anxiety/depression as two of its dimensions, in the second case. This could have affected the modelling results due to the potential for correlation, though the predictor variables were used as point assessments while the outcome variable was change on the EQ-5D Index.

Previous studies looking at predictors of QoL in head and neck cancer patients found that the presence of a feeding tube had the most negative impact on QoL, followed by medical comorbid conditions, presence of a tracheotomy tube, chemotherapy, and neck dissection. Hospital site, age, education level, sex, race, and marital status were also significant predictors of QoL [26]. Clinical predictors of pain included pre-treatment pain score, lower levels of education, neck dissection, feeding tube, xerostomia, depressive symptoms, taking more pain medication, less physical activity, and poor sleep quality [27]. Major predictors of change in the QOL of head and neck cancer patients from baseline to 1 year included depressive symptoms, alongside factors such as feeding tube placement, chemotherapy, and radiation therapy, as well as baseline smoking [28].

Although we also found that presence of anxiety/depression was a predictor of smaller improvements in QOL in all patient types, we only found that treatment factors (chemotherapy) were predictive of change in QOL in the colorectal patients. It is of course possible that withdrawal of chemotherapy from some patients over the study period could have influenced results, as chemotherapy can be associated with pain, though other studies have shown that chemotherapy can help to alleviate the pain caused by the tumour itself [29–32]. In the present study, we did not include an assessment of treatment side effects, though that could be of interest in future studies of this type.

Likewise, the present study was not intended to explore change in pain and QoL outcomes associated with specific treatment patterns, rather it was intended to provide an overall picture of change in pain and QoL outcomes achieved in routine clinical care, in a wide variety of clinical and geographical settings and including curative and palliative treatment with chemo- or radiotherapy. It also provides information regarding the proportion of patients who may still report extreme pain after 3 months of usual care as well as the proportion of patients who are likely to need treatment for anxiety and depression, which can be useful for planning care.

Strengths of the present study include the large sample size and the fact that patients were included from a total of 150 oncology units in a mix of settings from all regions of Spain. While it is not possible to ascertain how representative the sample is, its size and heterogeneity as well as the fact that it was drawn from Oncology Departments and pain clinics and therefore reflects the integrated management of patients with cancer, should provide a robust profile of pain and QOL outcomes for these patients in conditions of usual clinical practice.

Limitations of the study include the relatively short follow-up period and the fact that we did not collect information on all potentially relevant variables, such as educational level, sleeping, drinking or smoking, for inclusion in the regression models. In the present analysis, we also did not use the data collected at the one month visit. However, for practical reasons it was necessary to limit the number of study variables collected and those analysed for this paper. Another limitation is the fact that we did not perform an analysis of drop-outs from the study, which could be considered in future studies of this type. It should also be noted that some correlation between measures used in the study is bound to occur, as the EQ-5D already contains a pain dimension, so it would be surprising if improvements on the BPI did not to a certain extent predict improvements on the EQ Index. However, pain/discomfort is only one of the dimensions of EQ-5D and, as shown in Table 3, improvements on other dimensions were also observed. Likewise, the regression analysis indicates that other factors, not just improvement in pain, also play a role in the size of the QOL improvement observed.

In conclusion, we found that after 3 months of usual care in oncology units QoL and pain improved overall in patients with different types of cancer and moderate or severe pain at baseline, though results varied to some extent by cancer type. Improvements in QoL were associated with improvements in pain, whilst poor baseline performance status and the presence of anxiety and depression over the study period severely limited improvements in QoL. The study results provide an overall picture of change on relevant outcomes in routine clinical care in these patient groups in Spain and can provide a reference point to reflect on how such outcomes could be further improved in the future.

## Supporting information

S1 Dataset Dataset for this manuscript. (SAV)
